# Correlation analysis of serum levels of H19 and CRP levels and ulcerative colitis

**DOI:** 10.5937/jomb0-41359

**Published:** 2023-08-25

**Authors:** Aihua Wang, Yongkang Jiang

**Affiliations:** 1 Affiliated Danyang Hospital of Nantong University, The People's Hospital of Danyang, Laboratory Medicine, Danyang, China; 2 Danyang Hospital of Traditional Chinese Medicine, Laboratory Medicine, Danyang, China

**Keywords:** Ulcerative colitis, H19, CRP, ulcerozni kolitis, H19, CRP

## Abstract

**Background:**

To elucidate clinical applications of detecting serum levels of H19 and CRP in predicting the severity of ulcerative colitis (UC).

**Methods:**

Two hundred UC patients were recruited, and classified to mild/moderate group and severe group according to the Truelove-Witts grading system. Serum levels of H19 and CRP in UC patients were detected by turbidimetric inhibition immuno assay and qRT-PCR. Differences in serum levels of H19 and CRP between mild/moderate group and severe group were analyzed. By plotting ROC curves, the diagnostic potentials of H19 and CRP in UC were evaluated. Kappa conformance test was conducted to validate the conformance of detecting serum levels of H19 and CRP to clinical diagnosis of UC.

**Results:**

Serum levels of H19 and CRP were higher in UC patients of severe group than those of mild/moderate group. Their levels were both positively correlated to the severity of UC. High sensitivity (83.3%) and specificity (80.0%), as well as the maximum Youden index (0.633) were obtained at the cut-off value for H19 level of 2.755, and AUC was 0.8835. Meanwhile, Kappa coefficient (k) was 0.760 at the cut-off value for H19 level of 2.755, showing a high conformance to clinical diagnosis of UC. In addition, acceptable sensitivity (68.49%) and high specificity (85.83%), as well as the maximum Youden index (0.543) were obtained at the cut-off value for CRP level of 6.390 mg/L, and AUC was 0.8018. k was 0.435, showing an acceptable conformance to clinical diagnosis of UC based on serum level of CRP.

**Conclusions:**

Serum levels of H19 and CRP increase with the deterioration of UC. Detecting their serum levels has a consistent result to clinical diagnosis of UC, with a superior performance of H19 than that of CRP.

## Abstract (sr)

**Uvod:**

Cilj je bio da se razjasni klinička primena detekcije nivoa H19 i CRP u serumu u predviđanju težine ulceroznog kolitisa (UC).

**Metode:**

Regrutovano je dve stotine pacijenata sa UC i klasifikovano u blagu/umerenu grupu i tešku grupu prema Truelove-Vitts sistemu ocenjivanja. Nivoi H19 i CRP u serumu kod pacijenata sa UC su detektovani turbidimetrijskim imunotestom inhibicije i kRT-PCR. Analizirane su razlike u serumskim nivoima H19 i CRP između blage/umerene grupe i teške grupe. Primenom ROC krive procenjeni su dijagnostički potencijali H19 i CRP u UC. Kappa test usaglašenosti je sproveden da bi se potvrdila usklađenost detekcije nivoa H19 i CRP u serumu sa kliničkom dijagnozom UC.

**Rezultati:**

Nivoi H19 i CRP u serumu bili su viši kod pacijenata sa UC u teškoj grupi nego u blage/umerene grupe. Njihovi nivoi su bili u pozitivnoj korelaciji sa ozbiljnošću UC. Visoka osetljivost (83,3%) i specifičnost (80,0%), kao i maksimalni Youden indeks (0,633) dobijeni su na graničnoj vrednosti za H19 nivo od 2,755, a AUC je bio 0,8835. U međuvremenu, Kapa koeficijent (k) bio je 0,760 na graničnoj vrednosti za nivo H19 od 2,755, pokazujući visoku usklađenost sa kliničkom dijagnozom UC. Pored toga, prihvatljiva osetljivost (68,49%) i visoka specifičnost (85,83%), kao i maksimalni Youden indeks (0,543) su dobijeni na graničnoj vrednosti za nivo CRP od 6,390 mg/L, a AUC je bila 0,8018. k je bio 0,435, što pokazuje prihvatljivu saglasnost sa kliničkom dijagnozom UC na osnovu nivoa CRP u serumu.

**Zaključak:**

Nivoi H19 i CRP u serumu rastu sa pogoršanjem UC. Detekcija njihovog nivoa u serumu ima konzistentan rezultat u kliničkoj dijagnozi UC, sa superiornim performansama H19 od CRP-a.

## Introduction

Ulcerative colitis (UC) is a chronic, non-specific, inflammatory disease involving the colon and rectum [1]. Clinical manifestations of UC mainly include abdominal pain, diarrhea, hematochezia, fever, joint pain, etc [1] [2]. These symptoms can be sustained and repeatable, posing huge physical pain and mental burden for UC patients. It is generally considered that the involvement of genetic, environmental and immune factors during the progression of UC is complicated and undetermined [3]. It is necessary to clarify the pathogenesis of UC.

C-reactive protein (CRP) is an acute reactive protein, which is produced by the stimulation of IL-6 in hepatocytes [4]. It is the most widely used biomarker for monitoring the clinical activity of inflammatory bowel disease (IBD), even they are not always in a parallel relationship [5] [6]. For example, activities of systemic lupus erythematosus and UC are mainly regulated by cytokines, rather than IL-6 [7]. Since CRP is produced by IL-6 stimulation, it may not accurately reflect the severity of systemic lupus erythematosus and UC. In healthy people, the level of CRP remains low, which is remarkably enhanced following the acute immune response. Highly expressed CRP can be detected in patients with UC, acute myocardial infarction, rheumatoid arthritis, systemic lupus erythematosus, etc [8]. Therefore, CRP lacks clinical diagnostic accuracy for UC patients.

LncRNAs are functional RNAs that cannot be translated into proteins. There are a large number of lncRNAs in the genome, and they are extensively involved in biological activities through regulating gene expressions at epigenetic, transcriptional and post-transcriptional levels [9]. By microarray analysis and qRT-PCR detection, Wu et al. [10] uncovered 329 upregulated lncRNAs and 126 downregulated ones in the intestinal mucosa of UC patients. LncRNA H19 is a maternal imprinting gene, which is remarkably upregulated in the embryonic stage. Never - theless, it is downregulated only in the myocardium and skeletal muscles after birth [11]. Abnormally expressed H19 has a close relation to colorectal carcinoma, hepatocellular carcinoma and gastric cancer [12] [13] [14]. A relevant study demonstrated that H19 and vitamin D receptor signaling are involved in the progression of inflammatory diseases, and H19 in UC tissues contributes to the functional damage of the intestinal epithelial barrier [15]. Owing to the differential expressions of lncRNAs in UC process, they are believed to be promising biomarkers for diagnosis and treatment. This study aims to explore the diagnostic potentials of H19 and CRP in UC.

## Materials and methods

### Subjects

A total of 200 UC patients treated in xx hospital from xx to xx year were recruited. Diagnosis of UC lacks the gold standard, which requires to comprehensively take into considerations of clinical symptoms, laboratory, imaging, endoscopy and histo - pathological examinations. In the meantime, infectious and other non-infectious colitis should be excluded. Recruited UC patients were diagnosed based on the *Chinese consensus on diagnosis and treatment of inflammatory bowel disease (Beijing, 2018)* [16]. Suspected cases should be reexamined by endoscopy and histopathology 6 months later. UC patients were classified to mild/moderate group and severe group according to the Truelove-Witts grading system as follows [17]. Diagnostic criteria of severe UC: Number of defecations ≥ 6 times/d; Severe hematochezia; Pulse > 90 times/min; Temperature >37.8°C; Hemoglobin < 75% of normal level; ESR > 30 mm/1 h. Diagnostic criteria of mild UC: Number of defecations < 4 times/d; Mild hematochezia or none; Normal pulse, temperature and hemoglobin level; ESR < 20 mm/1 h. Symptoms of moderate UC were between those of mild and severe UC.

Exclusion criteria: (1) Patients with infectious colitis, ischemic enteritis, radiation enteritis, Crohn’s disease, etc.; (2) Patients with rheumatoid arthritis, allergic asthma, psoriasis, systemic lupus erythematosus and other immune-related diseases; (3) Patients with severe heart, liver or renal insufficiency, acute hemorrhage, malignant tumor and other diseases with bacterial infection. The study was approved by the hospital ethics committee, and all subjects have signed the informed consent.

### Laboratory examinations

The fasting venous blood of ulnar vein was collected within 24 h of admission, which was centrifuged at 1000×g for 20 min. The supernatant was collected and stored at -20°C. WBC (white blood cells), LY (lymphocyte count), NEUT (neutrophil count), MONO (monocyte count) and ESR (erythrocyte sedimentation rate) were detected using an automatic hematology analyzer. Serum level of CRP was detected using the BNII protein analyzer (Siemens, Germany).

### qRT-PCR

Total RNAs were collected using TRIzol (Invitrogen, Carlsbad, CA, USA), and qualified RNAs with OD260/OD280 of 1.8–2.0 were used for cDNA synthesis in a system containing 4 μL of 5×Primer Script Buffer, 10 μL of buffer, 1 μL of Primer Script RT Enzyme Mix, 2 μL of RT Primer and 3 μL of RNase-free ddH_2_O. Subsequently, qRT-PCR system was prepared as follows: 10 μL of 2×SYBR Premix Ex Taq^TM^II, 2 μL of cDNA, 1.6 μL of forward primer, 1.6 μL of reverse primer and 4.8 μL of RNase-free ddH_2_O. QRT-PCR was conducted at 95°C for 5 min, followed by 40 cycles at 95°C for 30 s, 65°C for 3 s and 60°C for 30 s. Relative level of H19 was calculated using 2-^∆∆Ct^ method. Primer sequences were: H19 F: 5’-TGATGACGGGTGGAGGGGCTA-3’, R: 5’- TGATGTCGCCCTGTCTGCACG-3’; GAPDH F: 5’- TGAACGGGAAGCTCACTGG-3’, R: 5’TCCACCACCCTGTTGCTGTA- 3’.

### Statistical analyses

Statistical Product and Service Solutions (SPSS) 22.0 (IBM, Armonk, NY, USA) was used for statistical analyses. Data were expressed as mean ± SD. Normally distributed measurement data were compared using the Student’s *t* test, and enumeration data were analyzed by chi-square test. ROC curves were plotted for assessing the diagnostic potentials. In addition, Kappa conformance test was conducted to validate the conformance of diagnostic values of H19 and CRP to clinical diagnosis of UC. *P*<0.05 was statistically significant.

## Results

### Symptoms and signs of severe and moderate/mild UC patients

A total of 73 recruited UC patients were severe cases, including 39 men and 34 women. Their average age was 47.35±6.97 years. In mild/moderate group, there were 127 UC patients, including 56 men and 71 women, with an average age of 46.4±6.35 years. Sex rate and age were comparable between groups (*P*>0.05). Notably, significant differences in BMI, course of disease and number of defecations were identified (*P*<0.05). In particular, lower BMI, longer course of disease and more times of defecations were detected in severe group (Table 1). Through laboratory examinations, no significant differences in WBC, LY and NEUT were examined, while MONO and ESR were higher in severe group (Table 1).

**Table 1:** Comparison of clinical and laboratory results of severe and mild/moderate UC patients. Note: Body mass index, BMI; white blood cells, WBC; Lymphocyte count, LY; Neutrophil count, NEUT; Monocyte count, MONO; Erythrocyte sedimentation rate, ESR

Variable	Mild/Moderate <br>UC (n=127)	Severe UC <br>(n=73)	t/χ^2^	*p*
Male (n)	56	39	1.618	0.240
Age (year)	46.4±6.35	47.35±6.97	-0.983	0.327
BMI (kg/m^2^)	23.74±3.21	22.48±3.02	2.730	0.007
Course of disease (year)	1.2±0.72	2.4±0.81	-10.836	<0.001
Number of stools (times/d)	3±0.41	7±0.71	-50.836	<0.001
WBC (×10^9^/L)	8.21±1.43	8.33±1.48	-0.564	0.573
NEUT (×10^9^/L)	5.87±1.15	6.15±1.26	-1.600	0.111
LY (×10^9^/L)	1.42±0.96	1.39±0.78	0.227	0.820
MONO (×10^9^/L)	0.21±0.071	0.33±0.1	-9.876	<0.001
ESR (mm/2h)	10±1.75	15±2.48	-16.640	<0.001

### Serum levels of H19 and CRP in UC patients

QRT-PCR data revealed higher serum level of H19 in severe UC patients than those of mild/moderate patients (Table 2). In addition, higher level of CRP was detected in serum of severe UC patients than the other group (Table 3). It is speculated that serum levels of H19 and CRP may be linked to the severity of UC.

**Table 2:** Comparison of serum levels of H19 between severe and mild/moderate UC patients.

Group	n	Mean±SD	t	*p*
Mild/Moderate <br>UC	127	1.60±0.49	-18.046	<0.001
Severe UC	73	3.09±0.67		
**Total**	**200**	**2.54±0.94**		

**Table 3:** Comparison of serum levels of CRP between severe and mild/moderate UC patients.

Group	n	Mean±SD	t	*p*
Mild/Moderate <br>UC	127	3.2±1.03	-14.153	<0.001
Severe UC	73	6.03±1.80		
**Total**	**200**	**5.90±1.89**		

### Clinical significance of H19 and CRP in UC

To ascertain the correlation between serum levels of H19 and CRP, and UC severity, correlation analysis was conducted. Based on the median serum level of H18 (2.54), OR=4.456 was calculated (95%CI=2.289–8.677) (Table 4). In the same way, OR of serum level of CRP was calculated as 2.508 (95%CI=1.364–4.612) (Table 5). The above data demonstrated that serum levels of H19 and CRP increased with the deterioration of UC.

**Table 4:** Correlation between serum level of H19 and severity of UC.

Group	n	H9		χ^2^	*p*	OR	95%CI
		< 2.54	≥ 2.54				
Mild/Moderate UC	127	68	59	20.787	<0.001	4.456	2.289–8.677
Severe UC	73	15	58				
**Total**	**200**	**83**	**117**				

**Table 5:** Correlation between serum level of CRP and severity of UC.

Group	n	CRP		χ^2^	*p*	OR	95%CI
		<5.90	≥5.90				
Mild/Moderate UC	127	66	61	8.967	0.003	2.508	1.364–4.612
Severe UC	73	22	51				
**Total**	**200**	**88**	**112**				

### Sensitivity and specificity of UC diagnosis based on H19 and CRP

**Figure 1:**
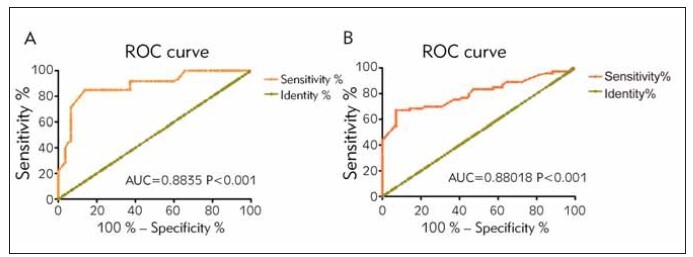
Sensitivity and specificity of UC diagnosis based on H19 and CRP. (A) High sensitivity (83.3%), specificity (80.0%), and the maximum Youden index (0.633) were obtained at the cut-off value for H19 level of 2.755, and AUC was 0.8835. (B) Acceptable sensitivity (68.49%), high specificity (85.83%), and the maximum Youden index (0.543) were obtained at the cut-off value for CRP level of 6.390 mg/L, and AUC was 0.8018.

Diagnostic potentials of H19 and CRP in UC were evaluated by plotting ROC curves. High sensitivity (83.3%) and specificity (80.0%), as well as the maximum Youden index (0.633) were obtained at the cut-off value for H19 level of 2.755, and AUC was 0.8835 (Figure 1A). In addition, acceptable sensitivity (68.49%) and high specificity (85.83%), as well as the maximum Youden index (0.543) were obtained at the cut-off value for CRP level of 6.390 mg/L, and AUC was 0.8018 (Figure 1B).

### Conformance of serum levels of H19 and CRP to clinical diagnosis of UC

To further validate the conformance of H19 and CRP levels to clinical diagnosis of UC, Kappa conformance test was conducted. κ was 0.760 at the cut-off value for H19 level of 2.755, showing a high conformance to clinical diagnosis of UC (Table 6). Similarly, κ was 0.435 at the cut-off value for CRP of 6.390, displaying an acceptable conformance (Table 7).

**Table 6:** Diagnostic potential of H19 in UC. Note: *P<0.05

H19	Clinical diagnosis		Kappa	χ^2^
	Mild/Moderate UC	Severe UC		
<6.390	110	6	0.760*	116.949*
≥6.390	17	67		

**Table 7:** Diagnostic potential of CRP in UC. Note: *P<0.05

CRP	Clinical diagnosis		Kappa	χ^2^
	Mild/Moderate UC	Severe UC		
6.390	91	19	0.435*	38.990*
≥6.390	36	54		

## Discussion

Inflammatory bowel disease (IBD) includes UC and Crohn’s disease (CD). Symptoms and signs of IBD are chronic and recurrent. Its incidence is annually enhanced in the world [18]. Symptoms of UC are non-specific, and typically occur intermittently. Current treatment of UC aims to induce mucosal healing and improve clinical symptoms [19]. However, monitoring the mucosal healing requires repeated endoscopy and even tissue biopsy. Precise, noninvasive biological markers for UC are urgently required [20].

CRP can distinguish between UC in active phase and resting phase. Besides, CRP is a reliable indicator that reflects mucosal healing [21]. It is noteworthy that the low specificity and high tendency of CRP variation remarkably limits the clinical application as a biomarker for UC [22]. Yoon et al. [23] analyzed the correlation between CRP and CDEIS (Crohn’s Disease Endoscopy Index Severity) according to 722 times of endoscopy examinations in 552 UC patients. They evaluated the sensitivity (50.5–53.3%) and specificity (68.7–71.3%) of CRP in assessing endoscopy examination of UC using 5 widely used scoring systems. It is concluded that CRP only has a mild correlation to CDEIS, and detection of serum level of CRP is considered as an adjuvant examination in UC patients.

LncRNAs are stably distributed in the body fluid and specifically expressed [24]. They have been proven as excellent biomarkers [25] [26]. LncRNA H19 contains 5 exons and 4 introns, which is highly conserved during evolution. It is mainly distributed in the cytoplasm, which is abnormally activated following tissue regeneration and tumorigenesis [11] [27]. Chen at al. [15] uncovered that overexpression of H19 causes the increase of intestinal epithelial permeability through downregulating TJ and VDR, which can be abolished by knockdown of miR-675-5p.

Our data revealed that serum levels of H19 and CRP were higher in severe UC patients in comparison to mild/moderate patients, verifying their proinflammatory properties. Subsequently, we found that H19 and CRP levels increased with the enhanced risk of UC severity. Diagnostic potentials of H19 and CRP in UC were confirmed through plotting ROC curves, and their conformance to clinical diagnosis of UC was later proven.

Collectively, H19 is a promising non-invasive biomarker for diagnosis and monitoring disease activity of UC. Nevertheless, several limitations should be mentioned. First of all, it was a single-center, retrospective cohort study. Second, differences of H19 and CRP between remission and active phase of UC were not analyzed. A multi-center, prospective, randomized controlled study with a large sample size is required to further validate our findings.

## Conclusion

Serum levels of H19 and CRP increase with the deterioration of UC. Detecting their serum levels has a consistent result to the clinical diagnosis of UC, and H19 has a superior performance than that of CRP.

## Dodatak

### Conflict of interest statement

All the authors declare that they have no conflict of interest in this work.
